# Identification of Clinically Approved Drugs Indacaterol and Canagliflozin for Repurposing to Treat Epidermal Growth Factor Tyrosine Kinase Inhibitor-Resistant Lung Cancer

**DOI:** 10.3389/fonc.2017.00288

**Published:** 2017-11-29

**Authors:** Hongjian Li, Christy Wing-Sum Tong, Yee Leung, Man-Hon Wong, Kenneth Kin-Wah To, Kwong-Sak Leung

**Affiliations:** ^1^Institute of Future Cities, The Chinese University of Hong Kong, Hong Kong, Hong Kong; ^2^Department of Computer Science and Engineering, The Chinese University of Hong Kong, Hong Kong, Hong Kong; ^3^Faculty of Medicine, School of Pharmacy, The Chinese University of Hong Kong, Hong Kong, Hong Kong

**Keywords:** epidermal growth factor tyrosine kinase inhibitor resistance, docking, drug repurposing, indacaterol, canagliflozin, *cis*-flupenthixol

## Abstract

In advanced lung cancer, epidermal growth factor tyrosine kinase inhibitors (EGFR TKIs) have extraordinary clinical efficacy. However, their usefulness is severely compromised by drug resistance mediated by various mechanisms, the most important of which is the secondary EGFR T790M mutation. The mutation blocks the binding of EGFR TKIs to the receptor kinase, thereby abolishing the therapeutic efficacy. In this study, we used our free and open-source protein-ligand docking software idock to screen worldwide approved small-molecule drugs against EGFR T790M. The computationally selected drug candidates were evaluated *in vitro* in resistant non-small cell lung cancer (NSCLC) cell lines. The specificity of the drugs toward the mutant EGFR was demonstrated by cell-free kinase inhibition assay. The inhibition of EGFR kinase activity and its downstream signaling pathways in NSCLC cells was shown by immunoblot analysis. The positive hints were revealed to be indacaterol, canagliflozin, and *cis*-flupenthixol, all of which were shown to induce apoptosis in NSCLC cells harboring the EGFR T790M mutation. Moreover, the combination of indacaterol with gefitinib was also found to produce synergistic anticancer effect in NSCLC cells bearing EGFR T790M. The observed synergistic effect was likely contributed by the enhanced inhibition of EGFR and its downstream signaling molecules.

## Introduction

One of the leading causes of cancer-linked deaths worldwide is attributed to lung cancer (1). The prognosis of advanced non-small cell lung cancer (NSCLC), a histologic subtype constituting more than 85% of all lung cancer cases, is very poor. Traditional cytotoxic chemotherapy is the major treatment option for advanced NSCLC, but treatment response is very minimal. Therefore, substantial research efforts have been made to identify novel targets for NSCLC.

As a member of the Erb family of receptor tyrosine kinases, the epidermal growth factor receptor (EGFR) protein plays a key role in the carcinogenesis of lung cancer. Upon stimulation of ligand, EGFR undergoes homo- or heterodimerization, which leads to autophosphorylation and subsequently turns on several downstream intracellular signaling pathways (including Ras/MAPK, PI3K/Akt, and Jak/STAT) to drive cancer growth and proliferation (2). In certain types of NSCLC, the EGFR gene is mutated and becomes constitutively active (i.e., exon 19 deletion or L858R mutation), leading to “addictive” oncogenic signaling (3). EGFR tyrosine kinase inhibitors (TKIs) target this “addictive” signaling and disrupt the downstream pathways to selectively kill cancer cells. However, the clinical efficacy of first-generation EGFR TKIs (gefitinib and erlotinib) is significantly hindered by both primary and acquired resistance (4). Primary resistance is mainly due to the activation of EGFR downstream molecules (KRAS mutation or PTEN loss), thereby bypassing the inhibition of EGFR activation by the drug. On the other hand, acquired resistance is mainly caused by the induction of a secondary EGFR T790M mutation, which reduces the affinity to EGFR TKIs. Aberrant regulation of parallel oncogenic pathways (MET amplification and HER2 mutation) has also been reported. Besides, overexpression of ABC efflux transporters (P-gp and ABCG2) also leads to acquired resistance (5). To this end, most EGFR TKIs are known substrates of these transporters (6, 7). In the clinic, gefitinib and erlotinib are only suggested for the treatment of NSCLC patients if they harbor activating EGFR mutations without the resistance mechanisms. At the effective dose in responsive patients, adverse effects from gefitinb and erlotinib are generally mild, the most frequent of which include rash and diarrhea. Intense research efforts have been made to search for alternative treatment options for patients who have resistance to EGFR TKIs (8, 9).

Numerous second-generation irreversible EGFR TKIs (e.g., neratinib, afatinib) have been developed to bind covalently to Cys-797 of the EGFR ATP-binding domain in order to bypass the most prevalent secondary EGFR T790M mutation (10). Second-generation irreversible EGFR TKIs exhibit potent inhibition of the isolated EGFR kinase in the low-nanomolar range. However, some of them (e.g., neratinib) are not effective against the resistant cells *in vivo* at a clinically achievable concentration (11, 12). Moreover, neratinib also exhibits severe dose-limiting toxicity of diarrhea due to its inhibition of wild-type EGFR (10). Recently, a few T790M-mutant-selective irreversible third-generation EGFR inhibitors have also been developed (13, 14). They exhibit potent growth inhibition effect on EGFR T790M-bearing NSCLCs *in vitro* at the low-nanomolar concentration (15). These inhibitors display high-EGFR T790M-mutant selectivity and irreversible binding patterns while sparing the wild-type EGFR activity, thus enhancing tumor selectivity while minimizing adverse effects (16–20). However, resistance to these third-generation EGFR TKI has already been reported, which is due to the emergence of another EGFR mutation C797S and various other mechanisms (21, 22). Most recently, the fourth-generation EGFR TKI (EAI045) has been developed to overcome the concomitant EGFR T790M and C797S mutations mediating resistance to third-generation inhibitor (23).

In this study, we aimed to identify new drug candidates for treating EGFR TKI-resistant lung cancer cells by employing the popular and efficient strategy of drug repurposing, given that new drug development and approval is often protracted and drug attrition rate is notoriously high. In contrast, repurposing drugs, which are already clinically approved with optimized dosing regimens and known side effect profiles, for other indications, has attracted a lot of attention. We hypothesized that clinically approved drugs may be exploited to circumvent EGFR TKI resistance and that they can be identified by *in silico* methods based on their predicted interaction with the T790M-mutant EGFR receptor.

Indeed, we have been working on *in silico* drug discovery and devised a couple of software tools and web servers (http://istar.cse.cuhk.edu.hk) for molecular docking, compound scoring and ranking, and molecular visualization. By using these *in silico* tools to screen a library of FDA-approved drugs, we have recently identified promising drug candidates exhibiting anticancer effect in both *in vitro* and *in vivo* assays for the treatment of liver, colon, and bladder cancers (24–26). Inspired by these successful recent cases, in this study we further improved the computational and experimental workflow to search for T790M-mutant EGFR inhibitors, which we believe will likely constitute a novel treatment of EGFR TKI-resistant lung cancer.

## Materials and Methods

### Structural Data Collection, Ensemble Docking, and Compound Selection

From the Protein Data Bank (PDB) (27), we collected 11 X-ray crystallographic structures of EGFR harboring the resistance-triggering T790M mutation in complex with a ligand (PDB IDs: 3W2O, 3W2P, 3W2Q, 3W2R, 4I22, 4RJ4, 4RJ5, 4RJ6, 4RJ7, 4RJ8, 5HG7). The EGFR structures alone were extracted from their respective complex with the co-crystalized ligand and water molecules detached, and then converted from PDB format to PDBQT format for later use by the docking software. According to the observation that the geometry of the binding site is usually proportional to that of the bound ligand, the cubic search space was placed at the geometrical center of the bound ligand, with the length, width, and height configured to be 30% greater than that of the bound ligand. Then the search space was further stretched by 4 Å in all three dimensions to create sufficient room for the drug molecules to translate and rotate inside and achieve the optimal conformation upon binding.

From the ZINC database (28), we collected the structures of approved drugs worldwide from three catalogs, namely DrugBank-approved, FDA-approved drugs (*via* DSSTOX), and the NCGC Pharmaceutical Collection (NPC). These compounds, having undergone a filtering and curation procedure, constituted a non-redundant set of 3,167 drugs that have been approved for clinical use by US (FDA), UK (NHS), EU (EMA), Japanese (NHI), and Canadian (HC) authorities. Likewise, the drug compounds downloaded in Mol2 format were also converted to PDBQT format for use by the docking software.

Having retrieved and preprocessed the necessary 3D structures, we then performed the prediction of binding conformations and binding affinities of the 3,167 compounds docking against the 11 T790M-mutant EGFR structures with the free and open-source molecular docking software idock v2.2.1 (29). Software settings were tweaked to make the conformational searching process substantially more exhaustive than the default settings, i.e., grid maps of atomic free energy were calculated with a high density of 0.08 Å for each receptor structure, and 256 conformational searching tasks were executed for each compound to increase the chance of finding the optimal binding pose. In addition, although up to nine putative binding conformations will be generated for each input compound under the default settings, we set to output only the docked conformation with the best idock score (which is always the first outputted conformation among the nine) because such pose had been formerly evaluated to be most likely closest to the crystal pose (30).

Following docking, the compounds were sorted in ascending order of their predicted binding free energy. Meanwhile, the more accurate scoring function RF-Score v3 was executed to rescore all the compounds (30), providing an additional and more reliable estimation of intermolecular binding strength, given the assumption that the compounds were correctly docked. Consequently, the top-scoring compounds would be those predicted to harvest both a low idock score (in terms of binding free energy) and a high RF score (in terms of binding affinity). These top-scoring compounds were visually inspected in the context of the receptor using the convenient web-based visualizer iview (31). Taking commercial availability into consideration, we purchased and consequently validated *in vitro* some of the potential compounds indicated from the *in silico* screening results (Table 1).

**Table 1:** The eight drug candidates shortlisted from *in silico* screening and later purchased and evaluated *in vitro*.

Drug name	ZINC ID	idock score	RF-score v3
Domperidone	19632603	−9.09	7.21
Ketanserin	537877	−9.05	7.10
Oxatomide	19632896	−8.74	6.78
Benperidol	19144237	−8.48	6.93
*Cis*-flupenthixol	29489118	−8.46	6.77
Canagliflozin	43207238	−8.35	6.69
Indacaterol	35801098	−8.35	6.70
Sildenafil	19796168	−7.28	7.26

*The idock score is an estimate of binding free energy in kcal/mol units. A more negative value implies a higher predicted binding stability. The RF-Score v3 is an estimate of binding affinity in negative log scale of Kd or Ki units. A more positive value implies a higher predicted binding affinity*.

### Cell Culture

Two NSCLC cell lines carrying specific EGFR mutations (HCC827: EGFR sensitizing E746_A750 deletion; H1975: resistance-causing EGFR T790M secondary mutation) were purchased from American Type Culture Collection (ATCC; Manassas, VA, USA). A normal human lung epithelial cell line BEAS-2B was also obtained from ATCC to investigate the potential toxic effect of the tested drugs. HCC827, H1975, and BEAS-2B cells were maintained in RPMI1640 medium supplemented with 10% fetal bovine serum, 100-U/mL streptomycin sulfate, and 100-U/mL penicillin G sulfate, and incubated at 37°C in 5% CO_2_.

### Growth Inhibition Assay

Growth inhibitory effects of various drug candidates on the cell lines were evaluated by the sulforhodamine B assay (32). Cells were seeded into 96-well tissue culture plates in 100 µL at a plating density of 3,000–5,000 cells/well, and allowed to incubate overnight. The cells were then treated with various drug candidates at a range of concentrations and allowed to incubate at 37°C in 5% CO_2_ for 72 h. Each drug concentration was tested in quadruplicate and controls were tested in replicates of eight.

### Evaluation of Drug Combination

Cells growth in 96-well plates were treated with either gefitinib or the repurposed drug alone or their combination in a fixed ratio of each drug in increasing concentrations. The drugs were added either simultaneously (24 h) or the tested drug (24 h) followed by gefitinib (24 h) or *vice versa*. The ratio of the two drugs in combination was initially determined according to the relative ratio of the IC_50_ values of the drug alone. For the three positive drug candidates, the range of concentration tested was as follows: indacaterol (10 µM–10 nM, in 1:1 serial dilution), canagliflozin (10 µM–10 nM, in 1:1 serial dilution), and *cis*-flupenthixol (2 µM–2 nM, in 1:1 serial dilution). Gefitinib was tested in the range of 10 µM–10 nM (1:1 serial dilution). The median-drug effect analysis method was used to evaluate the nature of the drug combination (33). Combination index (CI) was then calculated to assess the outcome of the drug combination at different fraction of cells affected (Fa) as described previously (33).

### Cell-Free Biochemical Kinase Inhibition Assay

Inhibition of tyrosine kinase signaling by the drug candidates identified was examined in a cell-free system by assessing the phosphorylation of a poly-EY (4:1 Glu, Tyr) peptide substrate (for EGFR) with recombinant kinases EGFR^wt^, EGFR^L858R^, or EGFR^L858R+T790M^ as described previously (34). Inhibition of the recombinant kinases by the drug candidates was evaluated by using the ADP-Glo Kinase assay kit according to the manufacturer’s instruction (Promega, Madison, WI, USA). Briefly, the drug candidates in a range of different concentration (indacaterol: 10 nM–10 µM; canagliflozin: 10 nM–10 µM; *cis*-flupenthixol: 2 nM–2 µM) were allowed to incubate with 4 ng of the recombinant kinases and 0.2 µg/mL of the poly-EY substrate at room temperature for 60 min. Also, 5 µL of ADP-Glo reagent was then added and incubation continued at room temperature for another 40 min. Afterward, 10 µL of kinase detection reagent was added and the mixture was allowed to incubate at room temperature for 30 min, before the measurement of luminescence by GloMax 20/20 Luminometer (Promega).

### Western Blot Analysis

Non-small cell lung cancer (HCC827, H1975) cell lines were treated with the tested drug candidates for the designated time (4 h). The cells were then harvested for Western blot analysis in lysis buffer (0.05-M HEPES pH 7.4, 0.15-M NaCl, 2-mM EDTA, 10% v/v glycerol, 1% v/v Triton X-100) supplemented with protease and phosphatase inhibitor cocktail (Thermo Scientific). Whole cell lysates were separated by SDS-PAGE and subjected to immunoblot analysis with the respective antibodies [total EGFR, phosphor-EGFR (Y845), phosphor-ERK1/2 (Thr177/Thr160), ERK1/2, phosphor-Akt, total Akt, and GAPDH (Santa Cruz Biotechnology, Santa Cruz, CA, USA)]. Primary antibody incubation was carried out at 4°C overnight in 5% bovine serum albumin/phosphate-buffered saline-Tween 20. Afterward, the membranes were incubated with HRP-conjugated donkey anti-mouse/anti-rabbit secondary antibody at room temperature for 1 h, and developed using the WesternBright Quantum chemiluminescence detection system (Advansta Corporation, Menlo Park, CA, USA). Anti-GAPDH antibody was used as the loading control (Santa Cruz Biotech, Santa Cruz, CA, USA). Digital chemiluminescence images were captured and quantified by using the FluorChem Q Imaging System (Alpha Innotech Corporation, Santa Clara, CA, USA).

### Annexin V Apoptosis Assay

Cells were grown on a 60-mm tissue culture dish at a density of about 5.0 × 10^5^ cells/well. They were treated for 48 h with the repurposed drug or its combination with 10-µM gefitinib. At the end of the treatment, both floating and attached cells were collected and washed twice with ice-cold phosphate buffer solution. The extent of apoptosis was determined by using the APC annexin-V apoptosis kit (BD Bioscience, San Jose, CA, USA) according to the manufacturer’s instructions. Cells positive for both annexin V and 7-AAD were considered apoptotic.

### Data Analysis and Statistics

All experiments were repeated at least three times. The results were conveyed as mean ± SD. One-way analysis of variance (one-way ANOVA) with Bonferroni’s multiple comparison test was used to compare the differences among various treatment groups. A confidence level of *p* < 0.05 was considered significant.

## Results

### Structure-Based Virtual Screening Provided Candidate Inhibitors for Repurposing

A total of 3,167 drugs approved for clinical use by worldwide authorities were individually docked to an ensemble of 11 crystal structures of the T790M-mutant EGFR kinase domain, and ranked in ascending order of their estimated binding free energy. The docking results (predicted binding poses of the compounds) can be openly visualized by accessing http://istar.cse.cuhk.edu.hk/idock/iview/?3W2R-dbap+fda+npc(The PDB ID 3W2R can be changed to 4I22, 4RJ8, or 5HG7 to view the other docking results using the corresponding protein conformation). The top-scoring compounds were manually assessed based on *in silico* estimations of binding strength, appropriate molecular weight and other drug-like properties, complementary matching of molecular shape, and some sense of intuition from a computational chemist’s experience. Based on commercial availability, eight high-scoring compounds (Table 1) were shortlisted and purchased for subsequent wet-laboratory investigations.

### Three Drug Candidates Inhibited Growth of NSCLCs

**Figure 1:**
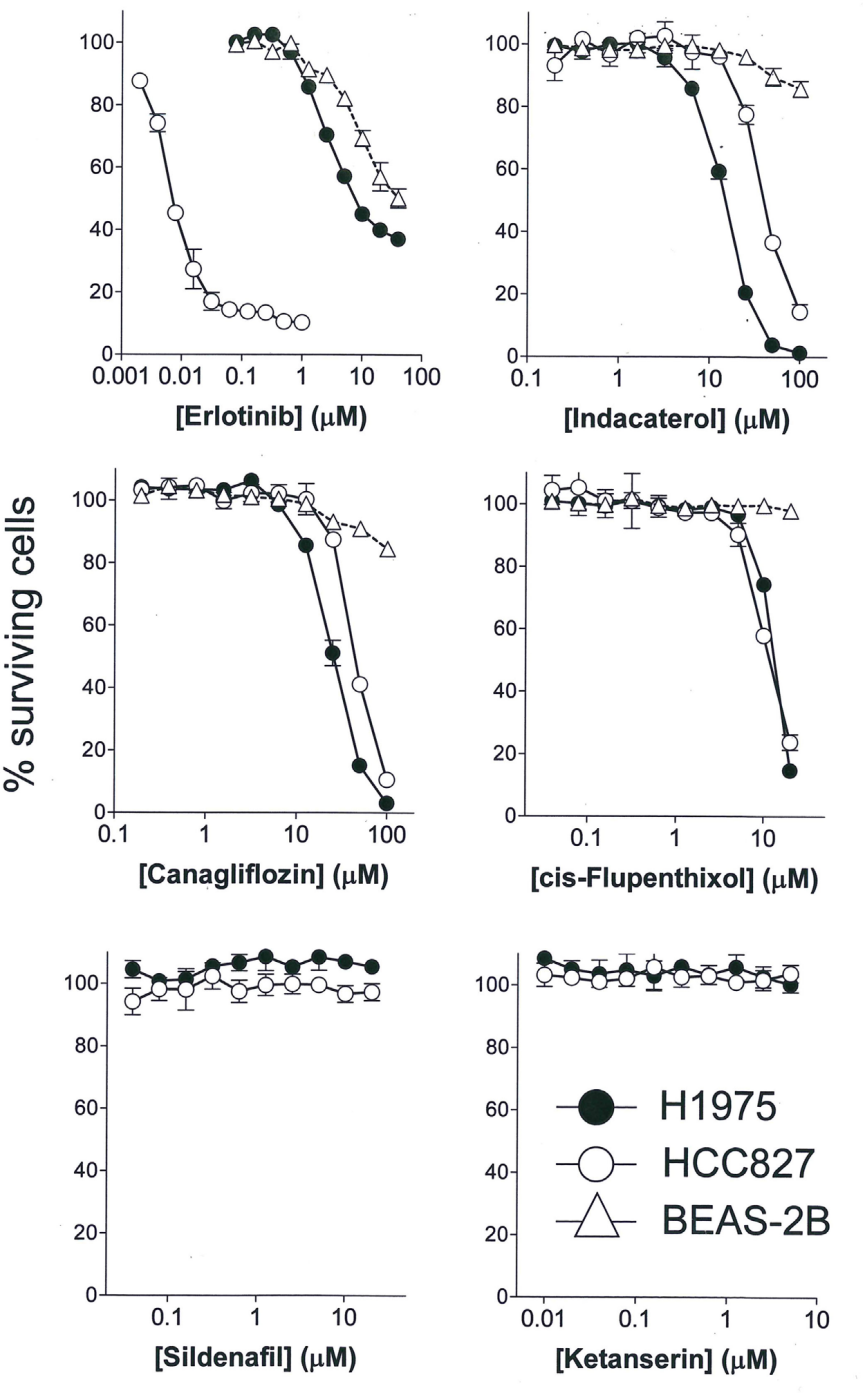
Cytotoxicity of the drugs tested. Cytotoxicity of three positive hits (indacaterol, canagliflozin, and *cis*-flupenthixol) and two negative drug candidates in the indicated cell lines was determined by the sulforhodamine B assay. A representative first-generation epidermal growth factor tyrosine kinase inhibitor (erlotinib) was also evaluated as control for comparison.

The anticancer activity of the drug candidates was tested in two NSCLC cell lines harboring EGFR sensitizing mutation (HCC827: E746_A750 deletion) or resistance-causing EGFR secondary mutation (H1975: L858R/T790M). HCC827 is extremely sensitive to erlotinib (first-generation EGFR TKI; IC_50_ = 6.1 ± 1.8 nM), whereas H1975 is more than 1,000-fold resistant to erlotinib (IC_50_ = 8.2 ± 2.1 µM) (Figure 1). Among the drug candidates tested, indacaterol, canagliflozin, and *cis*-flupenthixol were found to exhibit anticancer activity in both HCC827 and H1975 cells (Figure 1). Importantly, indacaterol and canagliflozin were both significantly more potent in inhibiting the growth of H1975 than HCC827 (indacaterol: 14.3 ± 2.2 µM in H1975 versus 41.5 ± 3.9 µM in HCC827; canagliflozin: 25.5 ± 3.1 µM in H1975 versus 45.4 ± 2.9 µM in HCC827). The other five drug candidates tested did not affect cell proliferation of HCC827 and H1975. Representative dose–response curves for two negative drug candidates (silfenafil and ketanserin) are shown in Figure 1. To assess the potential toxic effect of the drug candidates in normal lung tissue, cytotoxicity in a normal lung epithelial cell line BEAS-2B was also evaluated. While erlotinib was found to have an IC_50_ of around 20 µM, indacaterol, canagliflozin, and *cis*-flupenthixol only reduced cell viability by less than 20% at their highest concentration tested (indacaterol: 100 µM; canagliflozin: 100 µM; *cis*-flupenthixol: 20 µM).

### Positive Drug Candidates Inhibited the Kinase Activity of T790M EGFR in Cell-Free Assay

Since the positive drug candidates were identified by idock to interact with T790M EGFR, we hypothesize that their anticancer activity was contributed by their EGFR kinase inhibitory activity. The inhibitory effect of the positive drug candidates on the kinase activity of wild-type, L858R and L858R/T790M EGFR recombinant protein were evaluated in cell-free system. All of the three positive drug candidates were found to inhibit L858R and L858R/T790M EGFR to different extent (Table 2), presumably leading to the anticancer activity. Consistent with the preferential anticancer activity of indacaterol and canagliflozin in H1975 cells (harboring EGFR L858R/T790M resistance-causing mutation) over HCC827 cells (harboring the sensitizing EGFR E746_A750 deletion), the two drug candidates were found to inhibit EGFR L858R/T790M more significantly than EGFR L858R (Table 2). While the adverse effects of EGFR TKIs are known to be due to the inhibition of wild-type EGFR, the IC_50_ of the three positive hints in wild-type EGFR were at least two times (for *cis*-flupenthixol) higher than that in EGFR L858R/T790M.

**Table 2:** Inhibitory effect of tyrosine kinase activity by the tested drug candidates in cell-free *in vitro* kinase assay.

	IC_50_ (nM)				
Epidermal growth factor receptor	Erlotinib	Gefitinib	Indacaterol	Canagliflozin	*Cis*-flupenthixol
Wild type	0.84 ± 0.15 (70)	2.12 ± 0.25 (41)	792 ± 43.2 (3.4)	795 ± 49.6 (2.2)	186 ± 21.5 (2.2)
L858R	0.012 ± 0.001	0.052 ± 0.007	232 ± 36.5	368 ± 29.6	83 ± 5.2
L858R/T790M	220.7 ± 15.0 (18,392)	351.3 ± 22.4 (6,754)	103 ± 14.0 (0.4)	261 ± 24.4 (0.7)	94 ± 11.2 (1.1)

*Fold difference from L858R value in parenthesis. Two clinically used first-generation epidermal growth factor tyrosine kinase inhibitors (erlotinib and gefitinib) were also tested for comparison*.

### Positive Hints Inhibited the Autophosphorylation of EGFR and Its Downstream Pathway in NSCLC Cells

**Figure 2:**
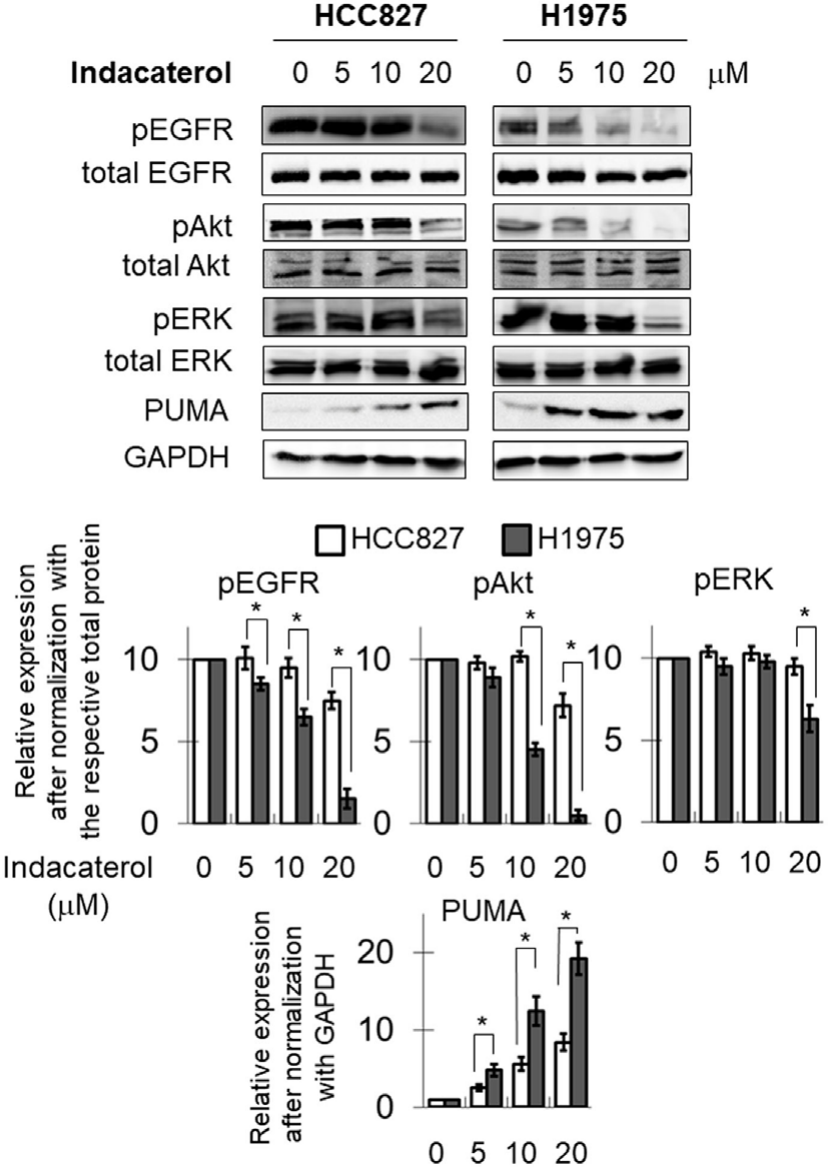
Western blot analysis showing the inhibition of the epidermal growth factor receptor (EGFR)–PI3K–Akt pathway by one of the three positive drug candidates (indacaterol) in both EGFR sensitizing mutation-bearing HCC827 cells and resistance-causing EGFR T790M mutation-bearing H1975 cells. The extent of inhibition of EGFR, Akt, and ERK phosphorylation is summarized after normalization with the respective total protein as bar graphs (lower panel). *p < 0.05, Student’s *t*-test, compared with the data in HCC827 cells. Cropped blots from different gels are grouped together for clear illustration. The full-length gels are shown in Figure S3 in Supplementary Material.

The inhibition of EGFR autophosphorylation by the three positive drug candidates were also examined in HCC827 and H1975 cells. Consistent with the data in the cell-free system, both indacaterol and canagliflozin were found to inhibit EGFR phosphorylation more potently in H1975 than in HCC827 cells (Figure 2; Figure S1 in Supplementary Material; albeit more obvious for indacaterol). On the other hand, *cis*-flupenthixol was found to inhibit EGFR phosphorylation similarly in both H1975 and HCC827 cells (Figure S2 in Supplementary Material). The phosphorylation status of Akt and ERK was chosen as marker to illustrate the inhibition of the EGFR downstream survival signaling pathways. Parallel to the inhibition of EGFR autophosphorylation by indacaterol and canagliflozin, these two positive drug candidates were also found to inhibit the phosphorylation of Akt and ERK more remarkably in H1975 than in HCC827 (Figure 2; Figure S1 in Supplementary Material).

### Positive Hints Induced Apoptosis in Concentration-Dependent Manner

**Figure 3:**
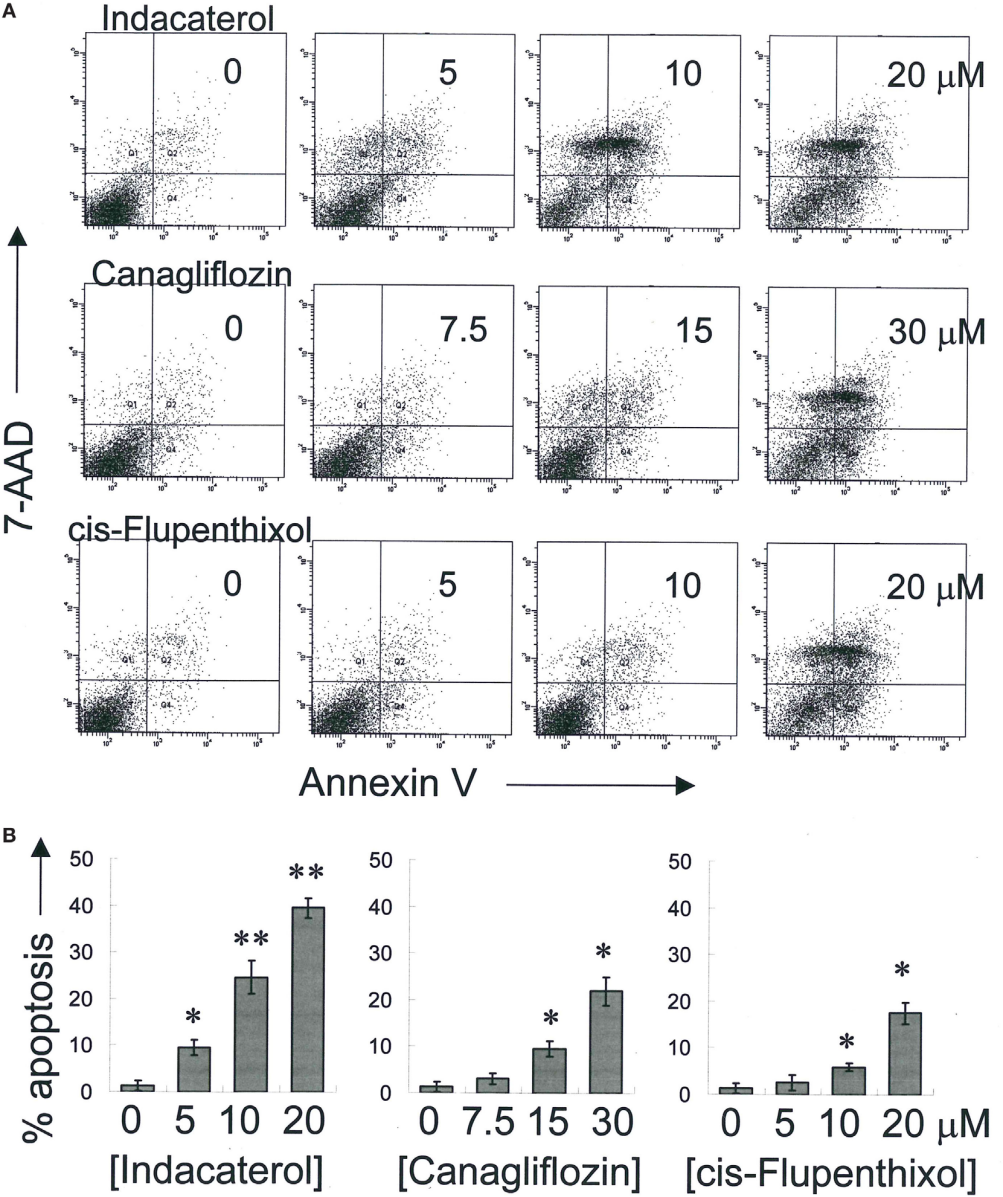
Induction of apoptosis by the three positive drug candidates in epidermal growth factor receptor T790M-bearing H1975 cells in a concentration-dependent manner. **(A)** Cells were exposed to the drugs at the designated concentration for 48 h before harvest for apoptosis assay. A representative set of data from three independent experiments is shown. **(B)** Summary of apoptosis assay data in **(A)** from three independent experiments. Data are presented in histogram as mean ± SD. *p < 0.05, **p < 0.01, Student’s *t*-test, compared with no treatment control.

Consistent with the anticancer activity found for indacaterol, canagliflozin and *cis*-flupenthixol in NSCLC cells, these drugs were also found to induce apoptosis in a concentration-dependent manner in the EGFR TKI-resistant H1975 cells (Figure 3). Since the key apoptotic effector PUMA is required for EGFR TKI inhibition-mediated apoptosis (35), the induction of PUMA by the repurposed drugs were also evaluated by Western blot analysis. Consistent with the more remarkable inhibition of EGFR in H1975 cells than in HCC827 cells by indacaterol and canagliflozin, both drugs were also found to give rise to more pronounced induction of PUMA in H1975 cells than in HCC827 cells (Figure 2; Figure S1 in Supplementary Material).

### Combination of Indacaterol and Gefitinib Synergistic in EGFR T790M-Bearing H1975 Cells

**Figure 4:**
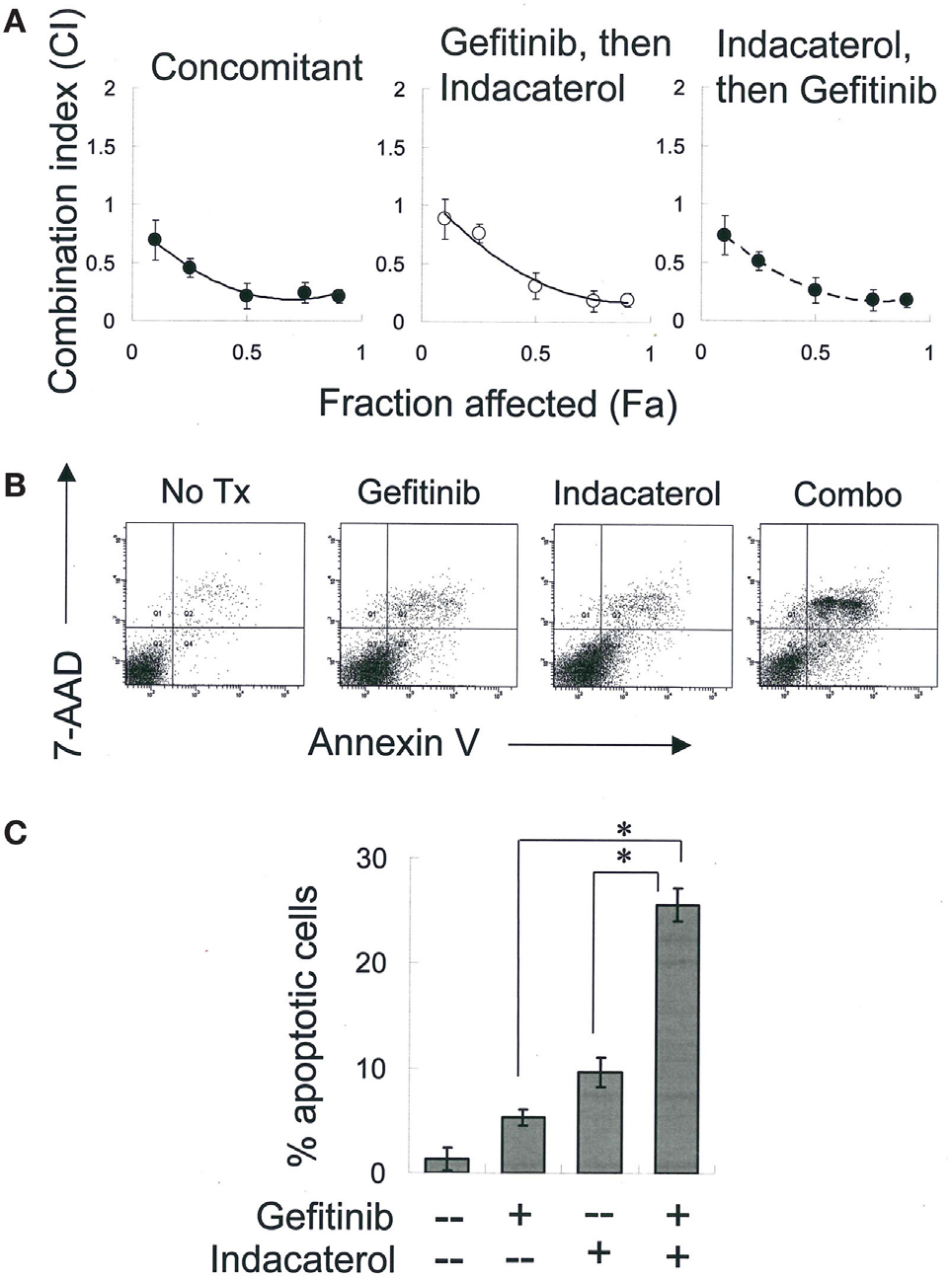
Combination of gefitinib and indacaterol giving rise to synergistic anticancer activity. **(A)** Combination index (CI) plot showing anticancer activity of combination of gefitinib and indacaterol in different sequence. CI values were plotted as a function of fractional growth inhibition (Fa). CI < 1, =1, and >1 suggest synergism, additivity, and antagonism, respectively. **(B)** Indacaterol sensitized H1975 cells to gefitinib-mediated apoptosis. Cells were treated with gefitinib alone (4 µM), indacaterol alone (5 µM), or their concomitant combination for 48 h before harvest for apoptosis assay. A representative set of data from three independent experiments is shown. **(C)** Summary of apoptosis assay data in **(B)**. Data are presented in histogram as mean ± SD. *p < 0.05, Student’s *t*-test.

Combinations of gefitinib (a representative first-generation EGFR TKI) with the most potent positive drug candidate (indacaterol) were evaluated in H1975 cells. Equipotent concentration ratio of gefitinib (10 nM–10 µM) and indacaterol (10 nM–10 µM) was used in the combination treatment. The concentrations were chosen so that each drug in the combination contributed equally to the overall anticancer effect. To determine whether the sequence of drug addiction affects the resulting drug combination effect, H1975 cells were treated concomitantly (for 24 h) with gefitinib and indacaterol, or 24 h with indacaterol, followed by drug-free washout and treatment with 24-h gefitinib, or *vice versa*. The CI was adopted as a measure of combination effect. The effects of the drug combinations are shown in the CI-fraction affected (Fa) plot (Figure 4A). For all drug combination sequence, a CI remarkably <1 was observed over a wide range of growth inhibition levels. At 50% growth inhibition level, a CI of around 0.3 was obtained, indicating a strong synergistic effect.

H1975 cells were treated with a combination of gefitinib (4 µM) and indacaterol (5 µM) concomitantly for 48 h, after which the extent of apoptosis was measured. While both gefitinib and indacaterol alone at the concentration tested only caused mild apoptosis (<10%), their combination was found to dramatically increase the proportion of apoptotic cells [27.8 ± 2.5% for drug combination versus 6.9 ± 1.2% (gefitinib alone) or 9.7 ± 1.8% (indacaterol alone); *p* < 0.01] (Figures 4B,C).

**Figure 5:**
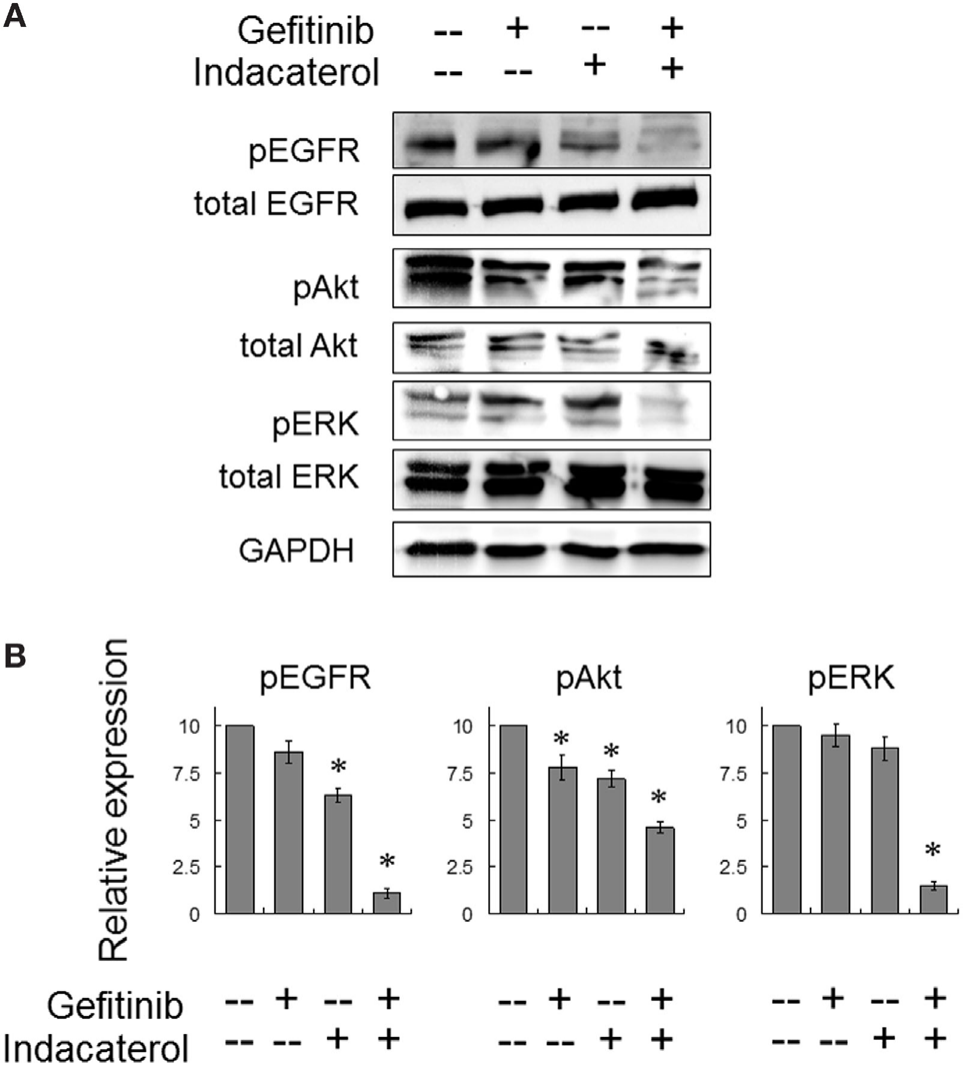
Combination of gefitinib and indacaterol giving rise to more pronounced inhibition of the epidermal growth factor receptor (EGFR)–PI3K–Akt pathway in EGFR T790M-bearing H1975 cells. **(A)** Cells were treated with gefitinib alone (10 µM), indacaterol alone (10 µM), or their concomitant combination for 4 h before harvest for Western analysis. A representative set of data is shown. Cropped blots from different gels are grouped together for clear illustration. The full-length gels are shown in Figure S6 in Supplementary Material. **(B)** Summary of the Western blot analysis from three independent experiments. Data are presented in histogram as mean ± SD. *p < 0.05, Student’s *t*-test, compared with no treatment control.

The activation of EGFR and its downstream signaling molecules (Akt and ERK) were also evaluated after concomitant combination of gefitinib (10 µM) and indacaterol (10 µM) for 4 h. While both gefitinib and indacaterol were only able to inhibit phosphorylation of EGFR, Akt, and ERK mildly, the drug combination was found to inhibit these signaling molecules more profoundly than the drugs alone (Figure 5).

### Putative Binding Conformation of Indacaterol to EGFR L858R/T790M Revealed by Molecular Docking

**Figure 6:**
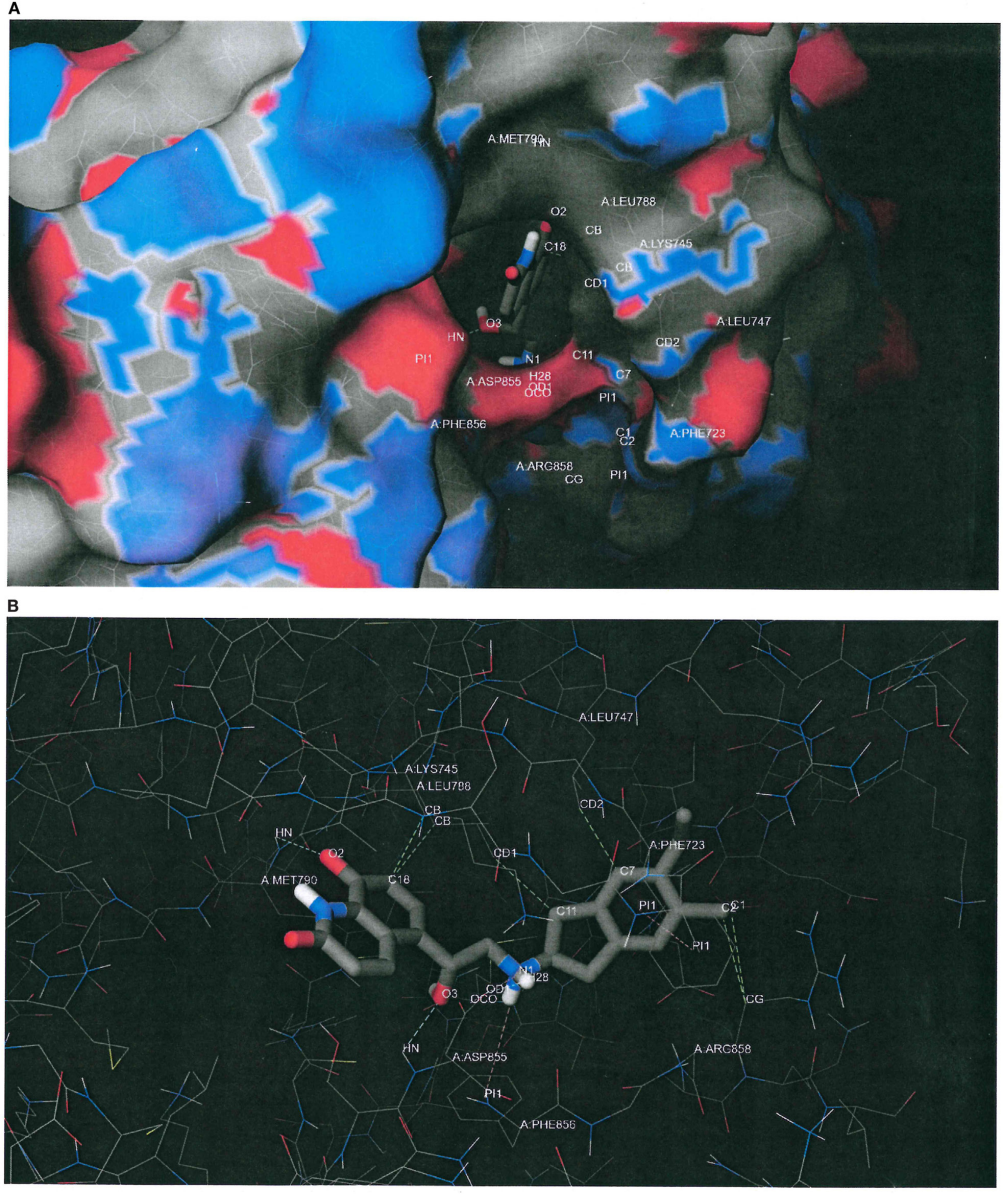
Putative binding conformation of indacaterol to epidermal growth factor receptor (EGFR) L858R/T790M active site (PDB ID 3W2R). **(A)** The putative binding pose of indacaterol on the macromolecular surface of EGFR L858R/T790M as predicted by the docking software. Carbons, oxygens, nitrogens, and sulfurs were rendered in gray, red, blue, and yellow, respectively. It can be seen that the indacaterol molecule was buried deep down in the binding pocket, with an oxygen atom acting as a hydrogen-bond acceptor and creating a hydrogen bond with the donor atom of the backbone of “gatekeeper” MET790. **(B)** The same predicted conformation of indacaterol upon binding to EGFR L858R/T790M, but with the macromolecular surface hidden in order to better inspect the putative intermolecular interactions, depicted by dashed lines. In addition to the hydrogen bond with MET790, indacaterol was predicted to establish two hydrogen bonds with ASP855 (cyan color), a salt bridge with ASP855 (purple color), a parallel displaced π stacking with PHE723 (pink color), a cation–π interaction with PHE856 (pink color), and some hydrophobic contacts with LYS745, LEU747, LEU788, and ARG858 (green color).

Figure 6A plots the macromolecular surface of EGFR L858R/T790M as well as the putative binding pose of indacaterol predicted by the docking software. Carbons, oxygens, nitrogens, and sulfurs were, respectively, rendered in gray, red, blue, and yellow. It can be seen that the indacaterol molecule was buried deep down in the binding pocket, with an oxygen atom acting as a hydrogen-bond acceptor and creating a hydrogen bond with the donor atom of the backbone of “gatekeeper” MET790. We hypothesize that the formation of the hydrogen bond might possibly permit the indacaterol molecule to be inserted into the binding cavity and therefore explain its specificity to EGFR T790M expressing cancer cells.

Figure 6B shows the same predicted conformation of indacaterol upon binding to EGFR L858R/T790M, but with the macromolecular surface hidden in order to better inspect the intermolecular interactions, depicted by dashed lines. In addition to the hydrogen bond with MET790, indacaterol was estimated to establish two hydrogen bonds with ASP855 (cyan color), a salt bridge with ASP855 (purple color), a parallel displaced π stacking with PHE723 (pink color), a cation–π interaction with PHE856 (pink color), and some hydrophobic contacts with LYS745, LEU747, LEU788, and ARG858 (green color).

## Discussion

The aim of the study was to identify clinically used drug candidates for repurposing to treat EGFR TKI-resistant NSCLC by *in silico* docking simulation and to validate the chosen drug candidates in cancer cell models.

Thanks to the rapid growth of number of 3D structures of large biological molecules deposited to the freely available PDB (27), structure-based virtual screening by protein-ligand docking has become a widely used computational method to quickly identify potential small-molecule inhibitors of protein targets of therapeutic interest. From the perspective of macromolecular targets, popular proteins such as kinases usually have more than one experimentally solved 3D structure available. These multiple structures of the same protein provide an opportunity to mine additional knowledge of structural similarity and variability using an approach called ensemble docking, which, in its simplest implementation, estimates the binding affinity of a compound by docking it to a desired binding pocket of multiple protein structures and averaging the individually predicted binding affinities. Hence, the top-scoring compounds would likely possess the capability of binding to diverse conformations of the protein. From the perspective of small-molecule compounds, approved drugs represent an attractive dataset to screen, as they have been clinically proved to be safe for human use, and are often well studied and well annotated. Finding novel therapeutic indications for already approved drugs is commonly referred to as repurposing or repositioning. The rationale is that a drug typically acts on more than one target and may exhibit previously unknown activities due to promiscuous off-target interactions explaining efficacy or side effects. This approach is substantially faster and cheaper with a lower attrition rate than developing new drugs, as all preclinical developments and toxicity tests prior to phase-II clinical trial can be skipped.

Encouragingly, the powerful synergy of drug repositioning combined with *in silico* ensemble docking has been demonstrated in two recent publications (24, 25), where, by targeting cyclin-dependent kinase 2 (CDK2), two FDA-approved drugs fluspirilene and adapalene have been rediscovered as anticancer agents *in vitro* and *in vivo* for the treatment of hepatocellular and colorectal carcinoma, respectively. Inspired by these successful previous cases, in this study we employed the same overall computational workflow with a number of improvements. First, the inclusion of small-molecule drugs from all major regulatory authorities worldwide for *in silico* screening, instead of limiting to the United States Food and Drug Administration data base, can greatly enhance the chance of obtaining successful hits. Second, we have combined the use of the docking software idock with our recently developed scoring function RF-Score v3 (30). While idock usually gives correct prediction of the binding poses of the drugs, the RF-Score-v3 scoring system is expected to provide an accurate prediction of the binding strength between the drug and the protein target. The additional information on the binding strength can then serve as an alternative classifier to help select a subset of positive hits from docking for consideration. Lastly, the use of a convenient web-based visualizer iview further facilitated the positive hit selection process by allowing complementary shape matching through molecular visualization (31). Therefore, the decision on the positive hits can be made by comprehensively considering different estimates of binding strength, appropriate molecular weight, as well as the binding conformation.

Based on the docking score and commercial availability, eight drug candidates were chosen from the *in silico* screening for *in vitro* evaluation in NSCLC-bearing wild-type EGFR, EGFR L858R (sensitizing mutation), or EGFR L858R/T790M (resistance-causing mutation). Three drugs were found to exhibit anticancer activity in the cancer cells and two of them were more potent in NSCLC-harboring EGFR L858R/T790M than those with EGFR L858R. Their higher specificity toward inhibiting EGFR L858R/T790M was further confirmed by cell-free biochemical kinase assay and Western blot analysis in the NSCLC cell models. The positive hits were also showed to inhibit the EGFR–PI3K–Akt signaling pathway and cause apoptosis in EGFR T790M-bearing NSCLC. Since the toxic effect of EGFR TKIs is known to be partially mediated by the inhibition of wild-type EGFR in normal cells, the finding that at T790M EGFR-inhibiting concentration (Table 2) the wild-type EGFR is not much affected suggests a milder adverse effect profile from the repurposing drugs identified.

Of note, one of the identified drug candidates, indacaterol, is a long-acting bronchodilator indicated for the treatment of chronic obstructive pulmonary disease (COPD). COPD and lung cancer are closely linked diseases with high prevalence of comorbidities in patients (36). Therefore, it is not unusual for an NSCLC patient to receive a medication to relieve his/her COPD symptoms. A salient point of identifying indacaterol is that it is administered as an inhalation powder and that it can achieve a locally high concentration in the lung. Although indacaterol has a higher IC_50_ on L858R/T790M EGFR-bearing NSCLC cells (~14 μM), as compared with a third-generation irreversible mutant-selective EGFR TKI (osimertinib) (~11 nM) (15), the concentration may be achievable for a locally administered drug. Moreover, due to the route of administration, drug–drug interaction is less of a concern. Furthermore, indacaterol may have other advantages compared with the third and fourth generations of EGFR TKIs such as osimertinib, which has only a short history of safe human use. Also, importantly, according to docking prediction, indacaterol does not target EGFR Cys-797, the mutation of which is a known mechanism of resistance for third-generation EGFR TKIs (21). Thus, indacaterol is likely less prone to drug resistance caused by the same mechanism. The drug candidates identified may be repurposed for treating NSCLC resistant to EGFR TKIs. Further research along this line may be able to identify more potent drugs for repurposing. The same *in silico* screening approach may be used to hunt for other clinically used drugs for repurposing to treat cancer cells resistant to other molecular targeted agents.

To overcome resistance to EGFR TKIs, besides developing new drugs, combination therapy is also a promising strategy. This approach aims at circumventing drug resistance through a so-called bypass signaling mechanism by targeting horizontal or vertical pathways or both. Interestingly, the repurposing drug indacaterol identified also gave rise to pronounced synergistic anticancer activity when used in combination with a model first-generation EGFR specifically in NSCLC cells harboring EGFR T790M TKI gefitinib. From the structural point of view, the anilinoquinazoline core of gefitinib forms a hydrogen bond with MET793 of the EGFR backbone, and its propylmorpholine tail picks up some non-specific interactions. In contrast, the recognition of indacaterol may be explained by different sets of intermolecular forces. According to the docking predictions, the indacaterol molecule was buried in the ATP-binding cavity, making a hydrogen bond to the gatekeeper MET790 backbone and two hydrogen bonds with ASP855, a parallel displaced π stacking with PHE723, a cation–π interaction with PHE856, and some hydrophobic contacts with LYS745, LEU747, LEU788, and ARG858 (Figure 6). We believed that the distinctly different sets of intermolecular forces between EGFR T790M and gefitinib versus indacaterol may allow a synergistic inhibition of the mutant kinase and thus enhanced anticancer activity by the drug combination. The combination of indacaterol and EGFR TKIs may be adopted as a novel means to circumvent resistance to EGFR TKIs. Further mechanistic investigation and animal studies are warranted to fully understand and optimize the beneficial drug combinations.

## Ethics Statement

This article does not contain any studies with human participants or animals performed by any of the authors.

## Author Contributions

KT, HL, K-SL, M-HW, and YL conceived and designed the study. KT, HL, and CT conducted experiments and analyzed data. KT, HL, and K-SL wrote the manuscript.

## Conflict of Interest Statement

The authors declare that the research was conducted in the absence of any commercial or financial relationships that could be construed as a potential conflict of interest. The reviewer LWD and handling Editor declared their shared affiliation.

## Supplementary Material

Figure S1 Western blot analysis showing the inhibition of the EGFR–PI3K–Akt pathway by one of the three positive drug candidates (canagliflozin) in both EGFR sensitizing mutation-bearing HCC827 cells and resistance-causing EGFR T790M mutation-bearing H1975 cells. The extent of inhibition of EGFR, Akt, and ERK phosphorylation is summarized after normalization with the respective total protein as bar graphs (lower panel). **p* < 0.05, Student’s *t*-test, compared with the data in HCC827 cells. Cropped blots from different gels are grouped together for clear illustration. The full-length gels are shown in Figure S4 in Supplementary Material.

Figure S2 Western blot analysis showing the inhibition of the EGFR–PI3K–Akt pathway by one of the three positive drug candidates (*cis*-flupenthixol) in both EGFR sensitizing mutation-bearing HCC827 cells and resistance-causing EGFR T790M mutation-bearing H1975 cells. The extent of inhibition of EGFR, Akt, and ERK phosphorylation is summarized after normalization with the respective total protein as bar graphs (lower panel). **p* < 0.05, Student’s *t*-test, compared with the data in HCC827 cells. Cropped blots from different gels are grouped together for clear illustration. The full-length gels are shown in Figure S5 in Supplementary Material.

Figure S3 Full-length gels for the Western blot data shown in Figure 2 (indacaterol).

Figure S4 Full-length gels for the Western blot data shown in Figure S1 in Supplementary Material (canagliflozin).

Figure S5 Full-length gels for the Western blot data shown in Figure S2 in Supplementary Material (*cis*-flupenthixol).

Figure S6 Full-length gels for the Western blot data shown in Figure 5 (combination of gefitinib and indacaterol).
