# Supplementary figures and images for: Identification of Clinically Approved Drugs Indacaterol and Canagliflozin for Repurposing to Treat Epidermal Growth Factor Tyrosine Kinase Inhibitor-Resistant Lung Cancer
Source: Front Oncol. 2017 Nov 29;7:288. doi: 10.3389/fonc.2017.00288 (PMC5712561; doi:10.3389/fonc.2017.00288)

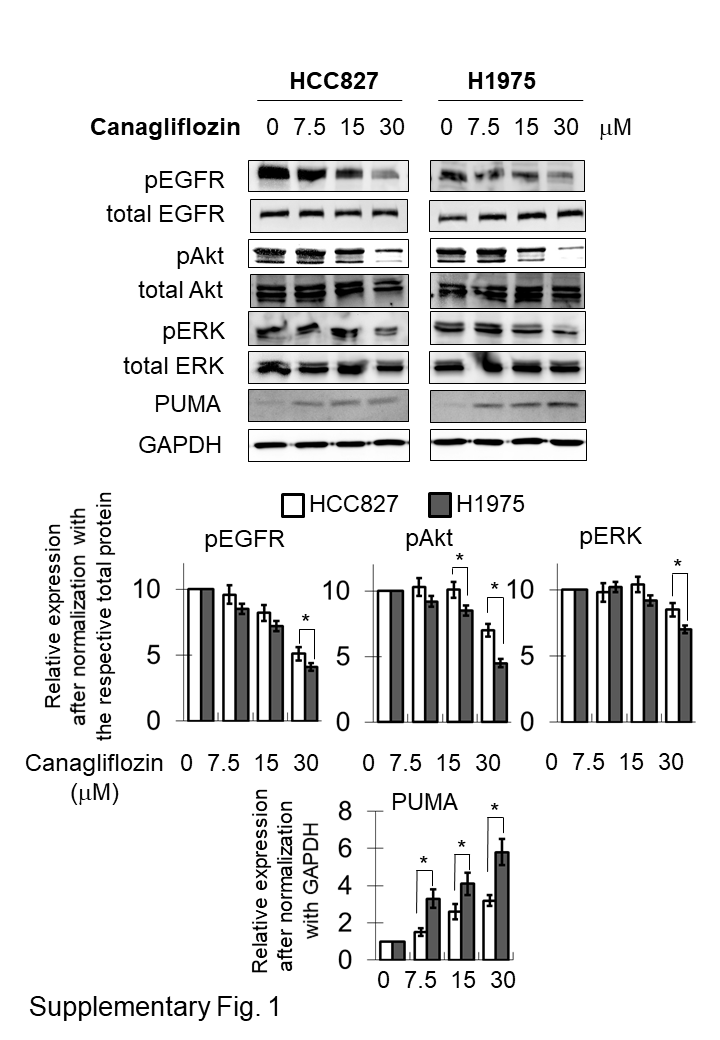

Supplement: Figure S1 — Western blot analysis showing the inhibition of the EGFR–PI3K–Akt pathway by one of the three positive drug candidates (canagliflozin) in both EGFR sensitizing mutation-bearing HCC827 cells and resistance-causing EGFR T790M mutation-bearing H1975 cells. The extent of inhibition of EGFR, Akt, and ERK phosphorylation is summarized after normalization with the respective total protein as bar graphs (lower panel). *p < 0.05, Student’s t-test, compared with the data in HCC827 cells. Cropped blots from different gels are grouped together for clear illustration. The full-length gels are shown in Figure S4 in Supplementary Material. [file Image_1.tif]

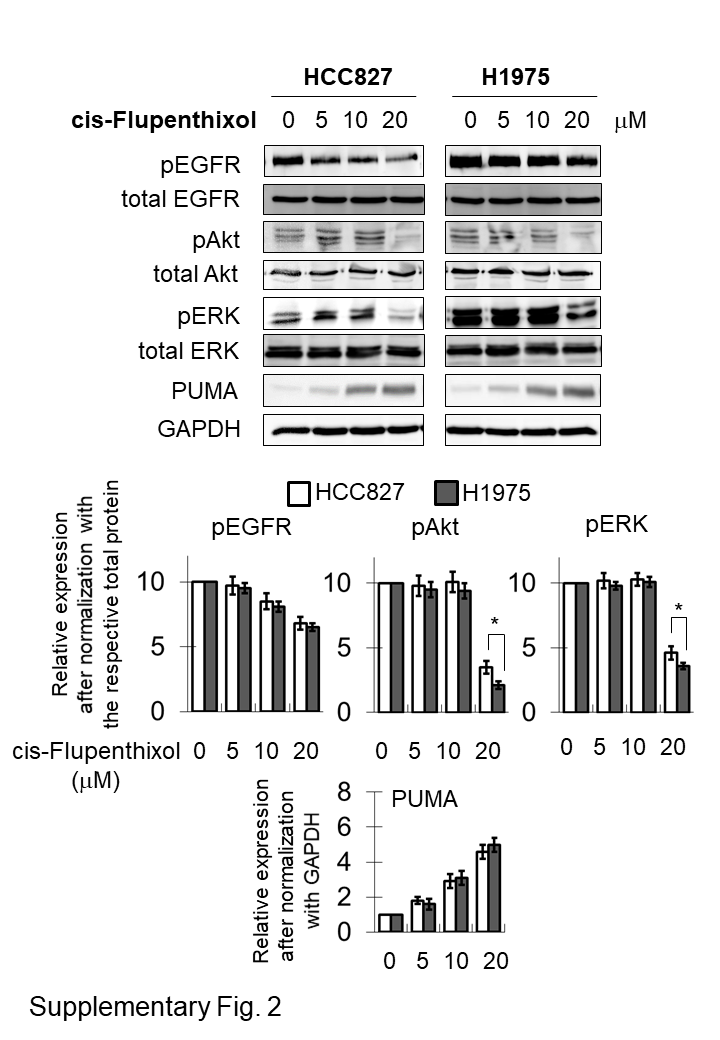

Supplement: Figure S2 — Western blot analysis showing the inhibition of the EGFR–PI3K–Akt pathway by one of the three positive drug candidates (cis-flupenthixol) in both EGFR sensitizing mutation-bearing HCC827 cells and resistance-causing EGFR T790M mutation-bearing H1975 cells. The extent of inhibition of EGFR, Akt, and ERK phosphorylation is summarized after normalization with the respective total protein as bar graphs (lower panel). *p < 0.05, Student’s t-test, compared with the data in HCC827 cells. Cropped blots from different gels are grouped together for clear illustration. The full-length gels are shown in Figure S5 in Supplementary Material. [file Image_2.tif]

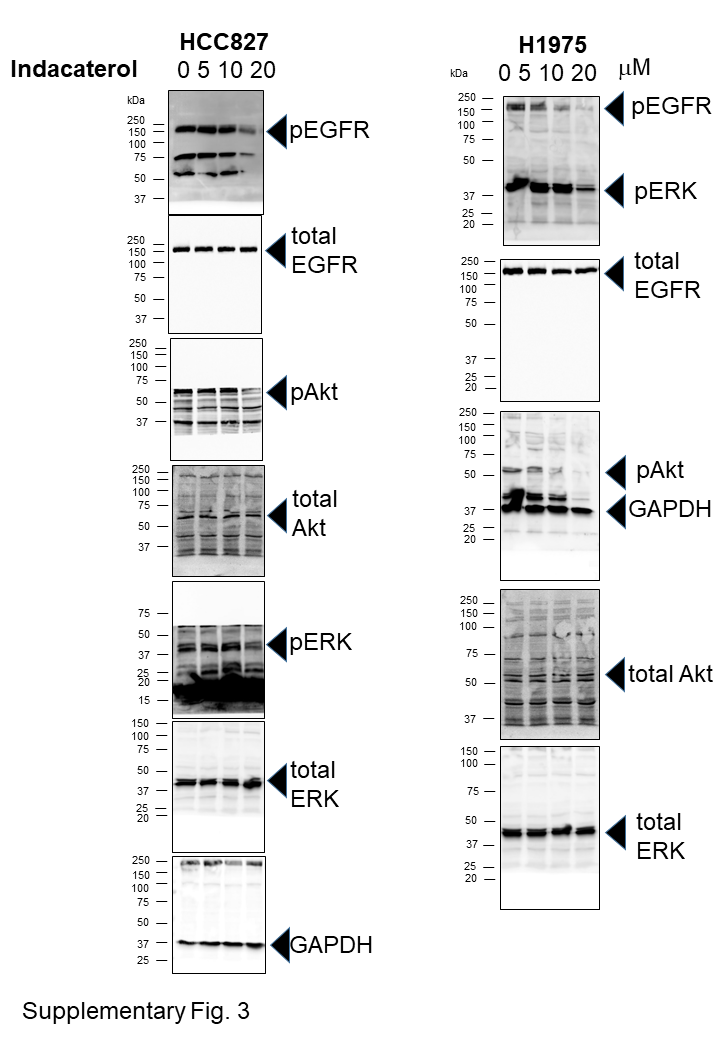

Supplement: Figure S3 — Full-length gels for the Western blot data shown in Figure 2 (indacaterol). [file Image_3.tif]

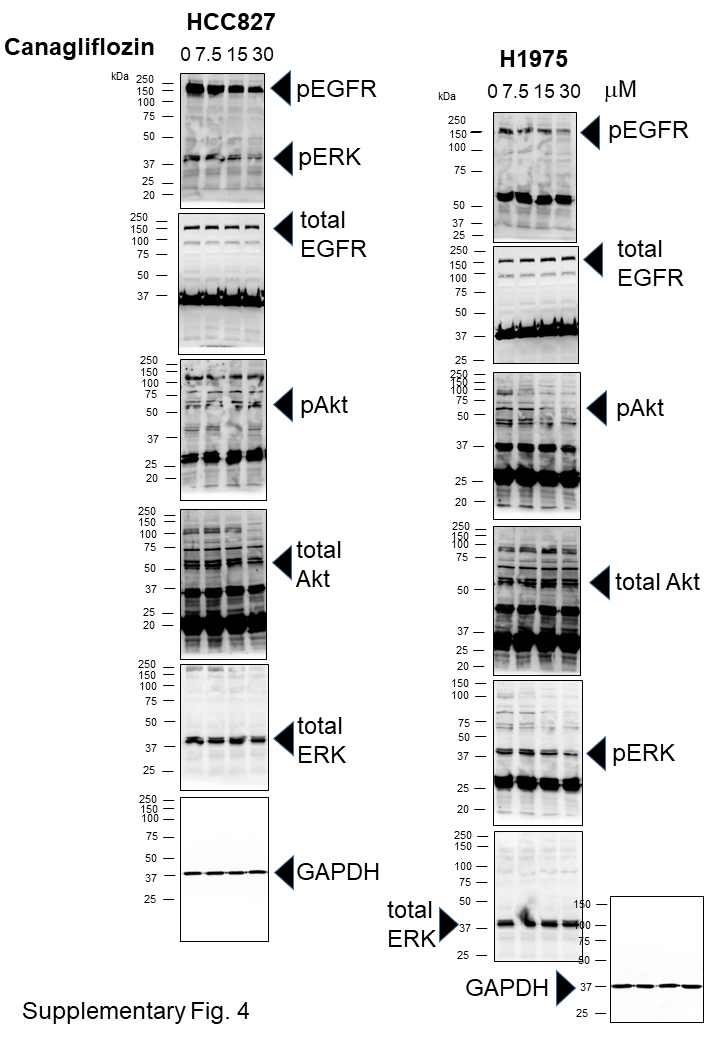

Supplement: Figure S4 — Full-length gels for the Western blot data shown in Figure S1 in Supplementary Material (canagliflozin). [file Image_4.tif]

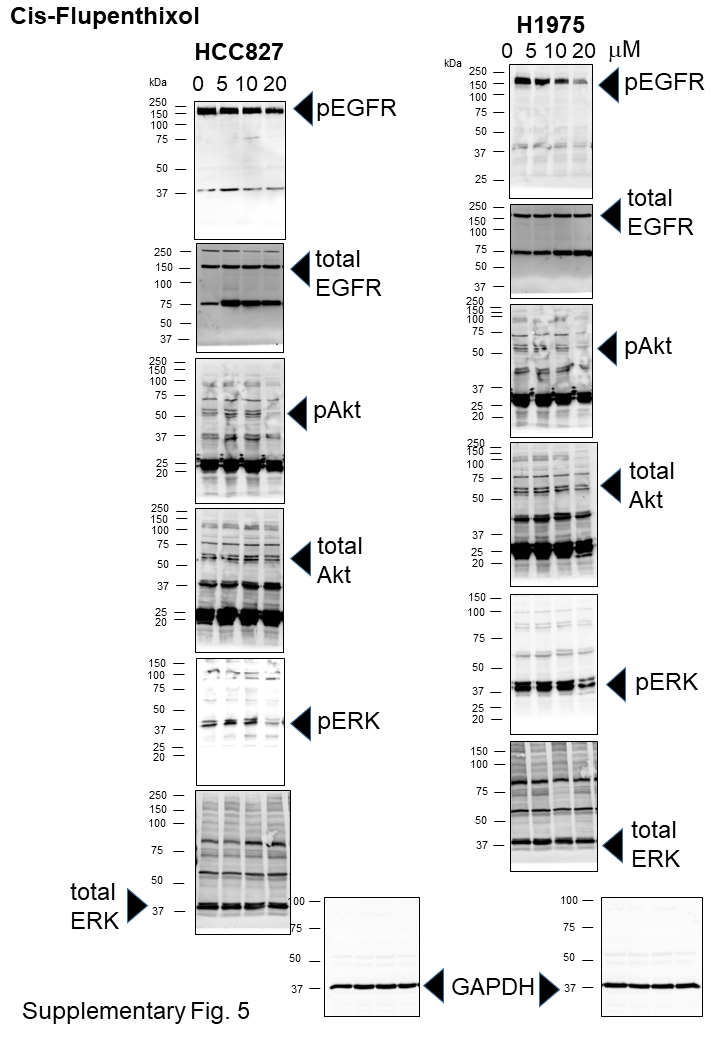

Supplement: Figure S5 — Full-length gels for the Western blot data shown in Figure S2 in Supplementary Material (cis-flupenthixol). [file Image_5.tif]

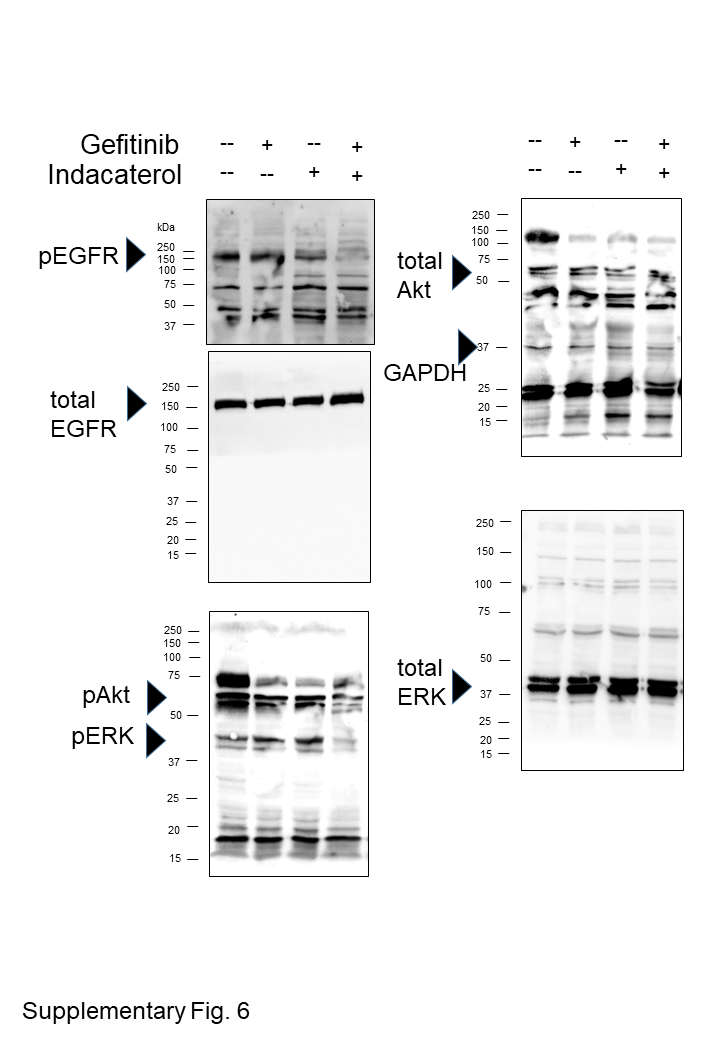

Supplement: Figure S6 — Full-length gels for the Western blot data shown in Figure 5 (combination of gefitinib and indacaterol). [file Image_6.tif]
